# A new form or a variant of SMD type A4

**DOI:** 10.1007/s13353-012-0094-0

**Published:** 2012-04-24

**Authors:** Ivo Marik, Olga Hudakova, Sarka Petrasova, Lukasz Kuszel, Malwina Czarny-Ratajczak, Kazimierz Kozlowski

**Affiliations:** 1 Ambulant Centre for Defects of Locomotor Apparatus, Olšanská 7, 130-00 Prague 3, Czech Republic; 2 Department of Anthropology and Human Genetics, Charles University, Vinicna 7, 128-43 Prague 2, Czech Republic; 3 Department of Medical Genetics, Poznan University of Medical Sciences, Grunwaldzka 55 paw. 15, 60-352 Poznan, Poland; 4 Department of Medicine, Tulane Center for Aging, Tulane University, School of Medicine, 1430 Tulane Ave., SL-12, New Orleans, LA 70112 USA; 5 Department of Medical Imaging, The Children´s Hospital, Westmead NSW, 2145 Sydney, Australia

## Introduction

Spondylometaphyseal dysplasias have been classified by Maroteaux and Spranger (1991) as well as by Duetting et al. (1998) based on severity of the changes in the femoral neck and metaphyses as well as vertebral abnormalities. SMD type A4 (SMDTA4) is characterized by severe changes of the femoral neck, marked metaphyseal abnormalities and ovoid, flattened vertebral bodies with an anterior tongue-like deformity (Maroteaux and Spranger 1991; Duetting et al. 1998). In addition to classical SMDTA4 characteristics, our patient has progressive scoliosis and lack of ossification of the capital femoral epiphyses at the age of 11 years. A genetic defect that cause SMDTA4 is so far unknown.

The Jansen type of SMD reported by Campbell et al. (2000), which is a phenotypic variant of Jansen metaphyseal chondrodysplasia, shares similar phenotypic features with our patient (Table 1). We decided to analyze the *PTHR1* gene encoding PTH/PTHrP receptor for parathyroid hormone related peptide (PTHrP) and parathyroid hormone (PTH), since mutations in this gene are a known cause of Jansen metaphyseal chondrodysplasia (Schipani et al. 1999).

**Table 1:** Phenotypic similarities and differences between patient with Jansen type of spondylometaphyseal dysplasia (Campbell et al., 2000) and reported patient

Phenotype	Patient with Jansen type of spondylometaphyseal dysplasia	Patient with new form or a variant of SMD type A4
Normal head and neck	+	+
Face	Dysmorphic	Normal
Large ears	+	+
Normal hearing and vision	+	+
Height	Short (-7 SD at 5.5 years)	Short (-9.2 SD at 6.5 years)
Short trunk	+	+
Normal lungs, heart, abdomen	+	+
Short extremities & rhizomelia	+	+
Genu valgum deformity	+	+
Relatively long hands & feet	+	+
Normal mental development	+	+

## Case report

**Fig. 1:**
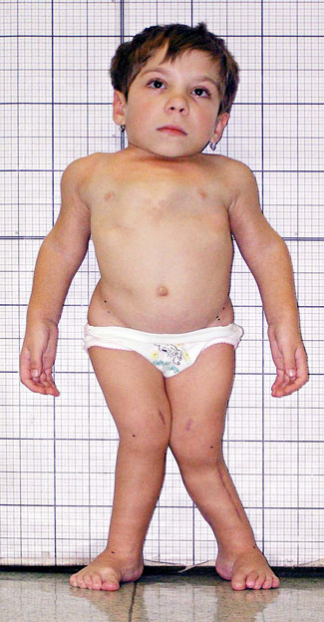
Photograph shows the 10-year-old patient. Note short stature (-10 SD = 80 cm), normal face, short trunk, rhizomelia, relatively long hands and feet and genu valgum deformity

The patient is a Caucasian female (Fig. 1), who was born to a 32-year-old mother and 34-year-old father after a third full term uneventful pregnancy. Her birth weight was 2700g, length 40 cm, head circumference 35 cm. The patient has two healthy half-siblings from the mother’s first marriage. Analysis of the family history did not reveal previous bone disorders in the other family members. Bone dysplasia was considered as a diagnosis due to the short stature of the newborn, however, no radiographs were taken at that time. The patient’s motor development was delayed; she started to walk at the age of 2 years. At the age of 3 years she was referred to the Ambulant Centre for Defects of Locomotor Apparatus in Prague. At that time her height was 62 cm (-8.8 SD), upper segment/lower segment = 39.7 cm (-.5 SD)/22.3 cm (-10.1 SD), arm span 63 cm, head circumference 48.6 cm (between 25-50 centile) and her weight was 8 kg. The patient had a genu valgum deformity and a waddling gait was observed. Her hands and feet were relatively long.

At the age of 4 years, patient was able to walk only for short distances. At that time she developed recurrent paroxysmal attacks of impaired consciousness with succession of muscular spasms. The cause of her seizures was hypertension secondary to a right dysplastic kidney. After right nefrectomy, her seizures disappeared and the blood pressure returned to normal. Bracing was used as a treatment for patient’s severe scoliosis at the age of 4 years.

**Fig. 2:**
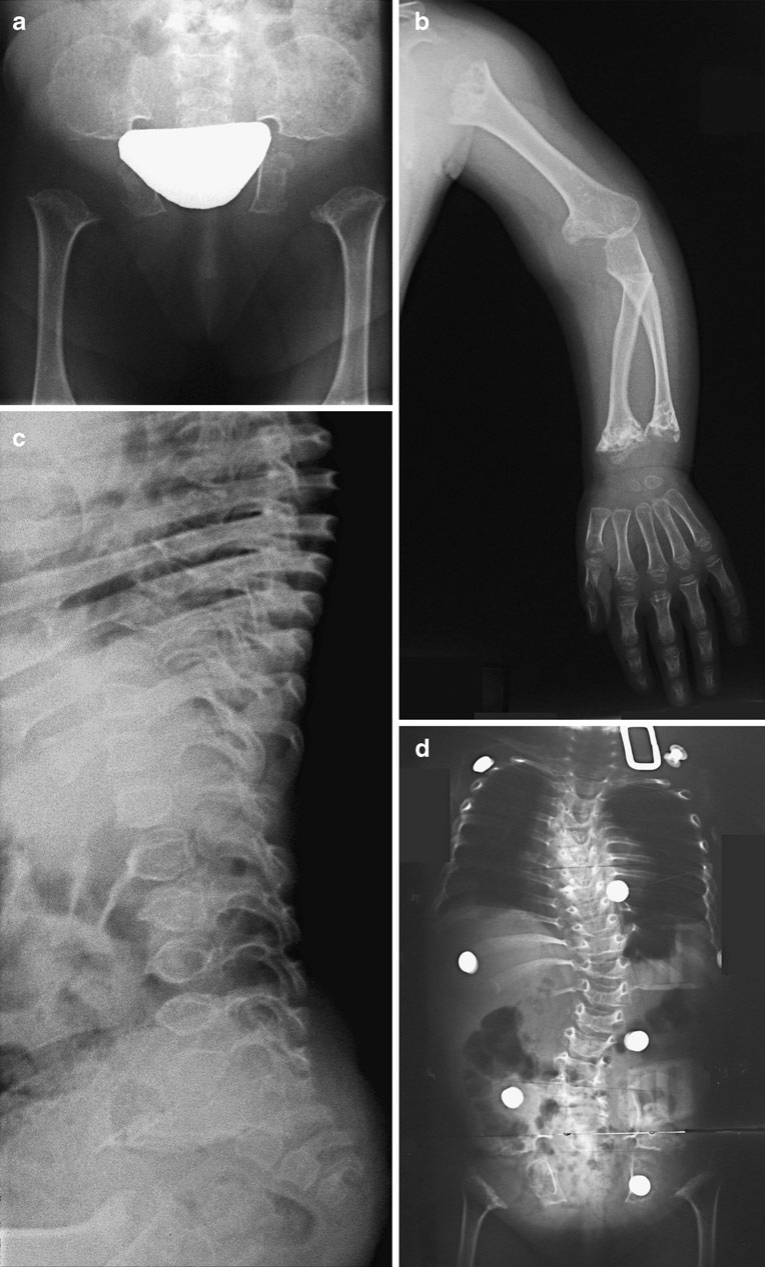
**a**-**d** Patient’s radiographs. **a**. Pelvis of the 2-year-old patient. There are round iliac wings, horizontal acetabular roof, short sciatic notches, broad ischia, very short and broad femoral neck and absent capital femoral ossification centres. **b**. Left upper extremity radiograph of the 3-year-old patient shows short humerus, marked metaphyseal involvement of the proximal humerus as well as distal radius and ulna and delayed carpal bone age. **c**. Lateral view of spine taken at the age 3 years demonstrates ovoid vertebral bodies with anterior tongue-like protrusion. **d**. **a**-**p** view of spine at the age 4 years reveals marked thoraco-lumbar scoliosis convex to the left

Radiographs taken between 2 and 6.5 years documented lack of ossification of the capital femoral epiphyses, severe metaphyseal changes, scoliosis convex to the left, and delayed carpal/tarsal bone age (Fig. 2a-d).

At 6.5 years her height was 72 cm (-9.2 SD) and her weight was 11 kg. The patient’s thickness of skin folds was normal and her muscle development correlated with the length of her extremities. Her mental development, vision and hearing were normal.

At the age of 9 years the patient’s height was 78.8 cm (-9.2 SD), the upper segment/lower segment ratio was 47 cm(-10.2 SD)/31.8 cm(-9.4 SD), span 86 cm, weight 13.3 kg and the head circumference 51.6 cm (33rd centile).

At 10 years her height was 80 cm (-10 SD) and her weight was 14.5 kg (Fig. 1). There was an increase of her spinal deformity (dextro-convex scoliosis of the thoracic spine and sinistro-convex scoliosis of the thoraco-lumbar spine with marked hyperextension), (Fig. 1). Her mobility was limited; she was walking by taking short steps.

**Fig. 3:**
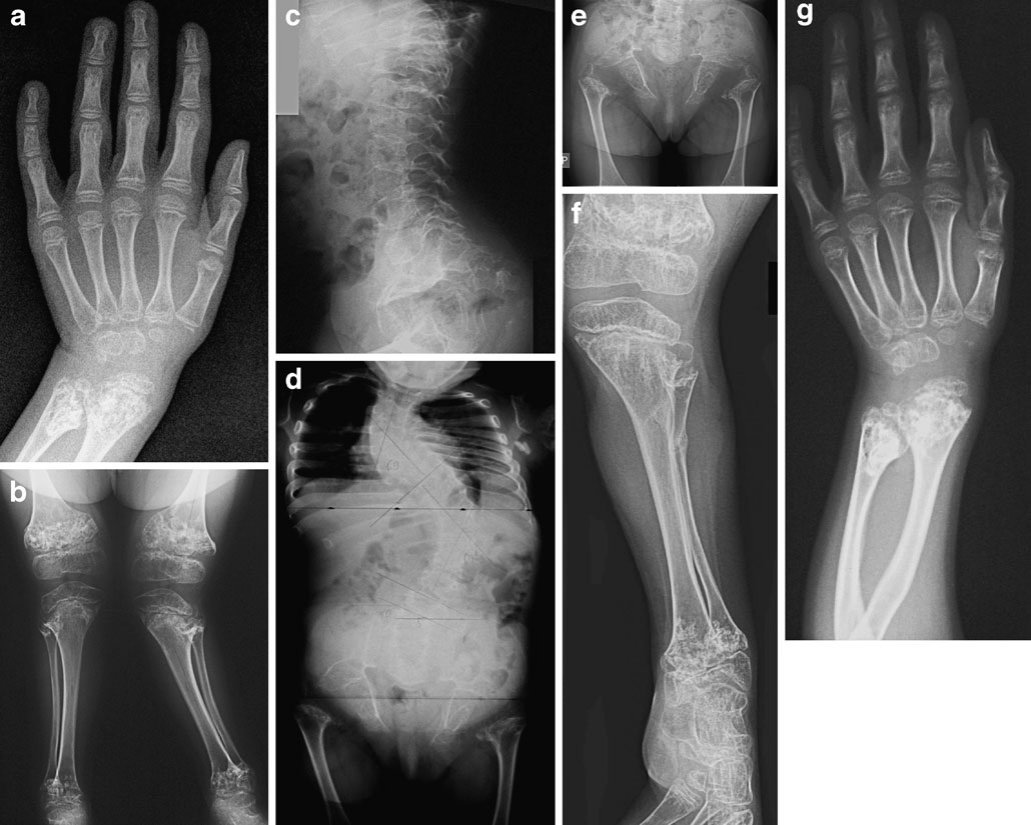
**a**-**g** Patient’s radiographs. **a**. Left hand of the patient at the age 5 years. There is marked metaphyseal involvement of the distal radius and ulna. Dissociation of the carpal bone age with absent ossification of the proximal carpal raw is demonstrated. Only four small carpal ossification centres are present. **b**. Knees of 8-year-old patient reveal marked metaphyseal involvement and severe coxa valga. **c**-**f** Patient at age 10 years. **c**. Lateral spine with lumbar lordosis and trapezoid shape of vertebral bodies. **d**. **a**-**p** view of spine that shows progress of spinal scoliosis in spite of 6 years of bracing. **e**. Pelvis. Note osteoporosis, absent capital femoral ossification and delayed ossification of the pubic bones. **f**. Left leg. Osteoporosis, severe metaphyseal changes and genu valgum deformity are visible. **g**. Left hand of 11-year old patient. The tubular bones of the hands are slightly affected. There is severe metaphyseal involvement of the forearm bones and dissociation of the delayed carpal bone age with absent ossification of the proximal carpal raw

Radiographs taken between 6.5 and 11 years of age documented progress of spinal scoliosis and increased genu valgum deformity. Ossification of the capital femoral epiphyses was absent and the carpal/tarsal bone age was delayed (Fig. 3a-g). Bilateral release of tractus iliotibialis and osteotomy of the left fibula, with corrective osteotomy of proximal metaphyses of the left tibia, were performed at the age of 8.5 years. At 9.5 years of age bilateral partial medial epiphyseodesis of distal femora was performed. Valgus deformity of the knees decreased after these surgeries.

## Methods and results

The laboratory tests showed normal routine blood and urine examinations. The serum levels of Ca, P and total alkaline phosphatase were normal as were the markers of bone turnover (bone alkaline phosphatase, osteocalcine and CTX – carboxyl-terminal telopeptide of collagen type I) and parathyroid hormone.

The patient had a normal karyotype (46,XX). Analysis of three regions of four common mutations (H223R, T410P, T410R, and I458R) in the *PTHR1* gene, did not reveal any changes in the sequence of this gene (Schipani et al. 1999; Bastepe et al. 2004). Analysis of the *PTHR1* gene was limited only to exons 7, 12 and 13 where the frequently mutated codons 223, 410 and 458 are located. Exons 7 and 12 were amplified by PCR with primers published by Czarny-Ratajczak et al. (2009). The following primers were used for amplification of exon 13 of the *PTHR1* gene: F5’-GGCAAGTCCAGATGCACTATG-3’ and R5’-GGAGGAACAAAGAAATAACAGG-3’. Sequencing of all PCR products was carried out directly using a dGTP BigDyeTM Terminator v3.0 Ready Reaction Cycle Sequencing Kit and an ABI Prism 3100 Sequencer (Applied Biosystems, Foster City, CA).

We detected via densitometry (DXA) low bone mineral density at the lumbar spine and at the whole body. Z-scores of our patient adjusted for age, sex and body size were less than -2.0.

## Discussion

We reviewed the literature and found no cases that fully resembled our patient who shares most phenotypic similarities with SMDTA4 patients described by Maroteaux and Spranger (1991), Duetting et al. (1998) as well as a patient reported by Campbell et al. (2000). Maroteaux and Spranger (1991) classified SMD accordingly to the femoral neck involvement, severity of metaphyseal changes and spinal abnormalities. SMDTA4 was mentioned in the classification of Maroteaux and Spranger (1991), however, without a detailed description.

Unlike other SMDTA4 patients reported in the literature, our patient has distinctive phenotypic features: short stature, congenital progressive scoliosis and progressive genu valgum deformity (Fig. 1). The radiographic changes include severe femoral neck involvement with absence of ossification of the capital femoral epiphyses at the age of 11 years, severe dysplasia of the knees with progressive valgus deformity and delayed carpal bone age (Figs. 2a-d and 3a-g). Lack of ossification of capital femoral epiphyses as well as progressive scoliosis have not been reported in SMDTA4. The carpal/epiphyseal dissociation with predominant involvement of the proximal carpal row observed in our patient was also noted in the second patient reported by Duetting et al. (1998). The patient presented by Campbell et al. (2000) as Jansen type of spondylometaphyseal dysplasia, also shares phenotypic features with our patient (Table 1). All other publications on SMD (Czarny-Ratajczak et al. 2009; Dieux-Coeslier et al. 2004; Goldblatt et al. 1991; Gustavson et al. 1978; Kozlowski et al. 1988; Kozlowski et al. 1976; Peeden et al. 1992; Shebib et al. 1991; Walters et al. 2004), characterize patients with phenotypes other than that of our patient, and a different pattern and severity of radiographic changes. The variety of SMD forms is most likely the consequence of mutations in different genes that are involved in cartilage development. Spondylometaphyseal dysplasias as well as spondyloepimetaphyseal dysplasias are very heterogeneous groups of bone dysplasias still explored at the molecular level, which in familial cases, frequently leads to identification of new candidate genes for these disorders. The unknown molecular background of SMDTA4 and lack of affected family members significantly limits the diagnostic options for sporadic patients with rare forms of SMD.
